# Cellulose-Based Fibrous Materials From Bacteria to Repair Tympanic Membrane Perforations

**DOI:** 10.3389/fbioe.2021.669863

**Published:** 2021-06-07

**Authors:** Bahareh Azimi, Mario Milazzo, Serena Danti

**Affiliations:** ^1^Department of Civil and Environmental Engineering, Massachusetts Institute of Technology, Cambridge, MA, United States; ^2^Department of Civil and Industrial Engineering, University of Pisa, Pisa, Italy; ^3^National Interuniversity Consortium of Materials Science and Technology (INSTM), Florence, Italy

**Keywords:** bacterial cellulose, eardrum, otitis media, myringoplasty, tympanoplasty, tissue engineering, modeling

## Abstract

Perforation is the most common illness of the tympanic membrane (TM), which is commonly treated with surgical procedures. The success rate of the treatment could be improved by novel bioengineering approaches. In fact, a successful restoration of a damaged TM needs a supporting biomaterial or scaffold able to meet mechano-acoustic properties similar to those of the native TM, along with optimal biocompatibility. Traditionally, a large number of biological-based materials, including paper, silk, Gelfoam^®^, hyaluronic acid, collagen, and chitosan, have been used for TM repair. A novel biopolymer with promising features for tissue engineering applications is cellulose. It is a highly biocompatible, mechanically and chemically strong polysaccharide, abundant in the environment, with the ability to promote cellular growth and differentiation. Bacterial cellulose (BC), in particular, is produced by microorganisms as a nanofibrous three-dimensional structure of highly pure cellulose, which has thus become a popular graft material for wound healing due to a number of remarkable properties, such as water retention, elasticity, mechanical strength, thermal stability, and transparency. This review paper provides a comprehensive overview of the current experimental studies of BC, focusing on the application of BC patches in the treatment of TM perforations. In addition, computational approaches to model cellulose and TM are summarized, with the aim to synergize the available tools toward the best design and exploitation of BC patches and scaffolds for TM repair and regeneration.

## Introduction

Bacterial cellulose (BC) is an extracellular polymer biosynthesized by bacteria from glucose and can be considered as the next-generation material due to its special properties, including high chemical purity (e.g., without any lignin and hemicellulose), excellent water uptake, degree of polymerization up to 8000, high tensile strength and thermal stability due to the crystalline nano-fibrillar structure coupled with the physico-chemical nature of BC (de Olyveira et al., 2011; Annabi et al., 2013; Singhsa et al., 2018). By virtue of its simpler, cheaper, and better environmentally friendly purification methods than those used for plant-based cellulose, BC has under consideration in several applications (Cacicedo et al., 2016). The 3D ultrafine fibrous and highly porous structure, high crystallinity index, biocompatibility, hemocompatibility, transparency, liquid absorbing capabilities and its poor solubility in physiological media, make the BC potentially useful for outstanding novel biomedical and pharmaceutical applications, including wound dressing (Kucińska-Lipka et al., 2015; Portela et al., 2019), skin engineering (Fu et al., 2012), cardiovascular (Leitão et al., 2016), ophthalmic (Levinson and Glonek, 2010; Kharaghani et al., 2020), skeletal systems (Torgbo and Sukyai, 2019; Wu et al., 2019; Pang et al., 2020), endodontics (Yoshino et al., 2013), drug delivery (Tamahkar et al., 2019; Weyell et al., 2019; Beekmann et al., 2020), and tissue engineering (Petersen and Gatenholm, 2011; Ul-Islam et al., 2015; Ullah et al., 2016; Qiao et al., 2018).

One remarkable field of application for BC is otology, and in particular the treatment of perforations of the Tympanic membrane (TM). Different factors, such as physical external trauma, purulent secretion or infections of the ear can result in eardrum perforation, which may bring to conductive hearing loss (CHL) and, possibly, long-term hearing damage (Mehta et al., 2006). Chronic otitis media (COM) is a recurrent disease of the middle ear sustained by an ongoing inflammatory process typically associated with unresolved and resistant bacterial infections, and is a leading risk factor for TM perforations. Other factors include frequent acute inflammation in the upper airways, genetic factors and local bacterial colonization. More than 32 million people worldwide are estimated to suffer from purulent COM, as reported by the World Health Organization (WHO), and half of them demonstrate permanent CHL, tinnitus, or dizziness (WHO, 2004). In COM, which can evolve into cholesteatoma, suitable medical and/or surgical therapy is required, leading to possible complications and recurrence (Louw, 2010). Depending on the extent of eardrum damage, different surgical approaches, including myringoplasty and tympanoplasty, are currently carried out using autologous tissue, such as fat for small perforations, or temporal fascia and cartilage for wide perforations (Aggarwal et al., 2006; Silveira et al., 2016). However, successful TM closure with optimal functional outcomes are not always obtained. Drawbacks of auto/allografts depend on their unspecific biological and mechanical properties that challenge the stability and functionality of the replacement. For example, a proper recovering of the hearing function is difficult to achieve since the mechano-acoustic properties of the currently used graft materials are not optimal for sound pick-up and transmission. Moreover, a long-term structural TM restoration may not be obtained since graft tissues are often resorbed, especially in chronically inflamed ears (Feenstra et al., 1984). Developing an efficient platform alternative to auto/allografts can be considered the next generation advancement in otology. To this purpose, it is fundamental to understand the factors influencing the success or failure of a repaired TM. Several biomaterial-based scaffolds and biomolecules have been evaluated for eardrum tissue engineering and different models have been developed to address a more accurate description of the eardrum shape (Kakehata et al., 2008; Kim et al., 2009; Mota et al., 2015; Anand et al., 2020; Li et al., 2020). For example, Anand et al. (2020) produced poly(ethylene oxide terephthalate)/poly(butylene terephthalate) (PEOT/PBT)-based TM scaffolds using a hybrid fabrication strategy combining electrospinning and additive manufacturing. They evaluated their efficiency as functional biomimetic TM replacements. Kakehata et al. (2008) have demonstrated that the application of chitin membrane in autologous serum eardrops therapy is a promising, safe, and feasible strategy for closing the TM perforations.

Bacterial cellulose has recently shown excellent properties in wound healing, as it has the ability to encourage cellular growth and differentiation that ultimately lead to faster healing (Sajjad et al., 2019). Recently, a few studies have investigated the efficiency of direct application of BC grafts for healing TM perforations (Kim et al., 2013; Biskin et al., 2016; Mandour et al., 2019). The aim of this review is to recapitulate the most recent advances and applications of BC for TM repair and replacement and give a future outlook on this emerging topic, including modeling.

## Bacterial Cellulose: Relevance for TM Pathology

Cellulose is the most abundant naturally occurring material on earth. It is the major component of a large number of materials including wood, algae, and cotton. It is a natural polymer of anhydro glucopyranose linked by the β (1→4) linkage (Zhou et al., 2020). BC is rich of Cellulose Iα (∼70%) (Newman, 1999; Nishiyama et al., 2002) while in plants Cellulose Iβ is more abundant in addition to hemicellulose and lignin (Wada et al., 1995; Nishiyama et al., 2008). The triclinic crystallinity of Cellulose Iα has been studied by Nishiyama with X-ray and neutron fiber diffraction (Nishiyama et al., 2002, 2008; Moon et al., 2011). Cellulose can be obtained from both a top–down approach, in which a desired size of cellulose can be produced from vegetal sources and a bottom-up approach, in which cellulose is biosynthesized from glucose using bacteria. Therefore, the cellulose structure and characteristics depend on the origin of the natural source and extraction process (Siró and Plackett, 2010).

**FIGURE 1:**
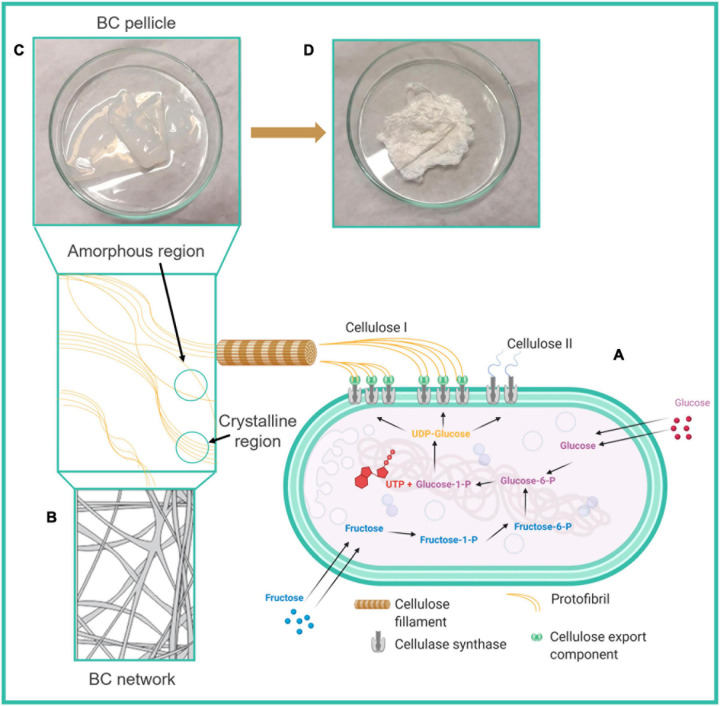
Schematic diagram of **(A)** the biosynthesis of bacterial cellulose (BC) from sugars, **(B)** 3D structure of BC network containing amorphous and crystalline regions, **(C)** BC pellicle, **(D)** dried BC. Reprinted with permission (Swingler et al., 2021) © Creative Commons Attribution License, 2021, MDPI.

Bacterial cellulose is an extracellular polymer produced by *Komagataeibacter xylinus* in four allomorphic forms, through the fermentation of sugars (Swingler et al., 2021) (Figure 1A). An alteration of culture conditions can induce such microorganisms to fabricate BC in different forms, such as sheets, pellets and films (Eslahi et al., 2020). Although BC and vegetal cellulose are both composed of linear homopolysaccharides conjugated by D-glucose units, there are slight differences in the crystal structure and microfibril arrangements, which result in quite different properties (Feng et al., 2015). Specifically, BC fibrils form a 3D structure network as shown in Figure 1B with an average diameter of 1.5 nm, having superior physicochemical and mechanical properties such as higher crystallinity, lower density, larger surface area, increased elasticity, flexibility and tensile strength than in the vegetal counterpart (Czaja et al., 2004; Prosvirnikov et al., 2018). In addition, BC is characterized by chemical purity (less than 10 w% of hemicellulose and lignin), thermal stability, good biocompatibility, liquid and gas permeability, non-pyrogenicity and hydrophilicity, which overall make it a good candidate for biomedical applications (Huang et al., 2014; Gorgieva, 2020). The method of fermentation, performed in static or dynamic conditions, influences the features of BC structures, namely, surface area, poral volume, and pore size. Water retention capacity is directly proportional to the surface area and pore volume of BC, whereas pore size influences the physical and mechanical properties of the material network (Swingler et al., 2021) (Figures 1C,D).

Bacterial cellulose nanofibrillar-based patches are excellent candidates for TM repair and regeneration. TM has peculiar anatomic features, which allow sound transmitting function (Fay et al., 2006; Mota et al., 2015; Milazzo et al., 2017, 2020). Human TM has a varying thickness of several tens to hundred microns and is composed of a tri-laminar tissue (Fay et al., 2005). The mid-layer is connective tissue proper, while the outer layers are epidermal or mucosal epithelia. The *pars tensa* represents the largest area of the TM. It is composed of collagen fibers responsible for the precise vibratory function of TM (Fay et al., 2006; Volandri et al., 2011; Milazzo et al., 2017).

Tympanic membrane perforation, both traumatic and infectious origin, is a very common clinical problem worldwide. As this tissue is suspended in between two air filled cavities, the outer ear canal and the tympanic cavity, once the defect takes place, the extent of the damage determines the capacity for self-repair. In partial perforations, the TM can regenerate spontaneously, while in large perforations that are supported by inflammation or chronic otorrhea, it cannot undergo spontaneous healing (Kim et al., 2013; Sakshi, 2017). In these cases, different types of graft materials are currently applied (Laidlaw et al., 2001; Ayache et al., 2003). However, they are not always efficient in TM closure. The larger the defect, the harder is its reconstruction with an autologous material, and long-term TM healing may not be obtained since the material integrity is challenged by the undergoing inflammatory processes (Feenstra et al., 1984). Temporalis fascia, which is the “gold standard” graft, requires massive manipulation and longer surgical times (Ayache et al., 2003). The paper patch graft technique has shown to guide TM cells, usually epithelial cells, to migrate from the borders of perforation to the patch, thus filling the gap in small and clean perforations. However, functional results can be suboptimal (Kim et al., 2009). Therefore, developing an efficient healing platform for TM perforation, as an alternative to currently used grafts, is considered the next goal in otology.

The nanofibrous structure of BC has the ability to enhance tissue regeneration, because it resembles the natural structure of an extracellular matrix (ECM). As such, it can mimic ECM behavior and guide the proliferation of tympanic cells to reach the rupture site (Costa et al., 2012; Kim et al., 2013). Notably, BC has the ability to support cell growth of all three layers of the normal eardrum (i.e., epidermal, connective, and mucosal layers) and thus the potential to form a full-thickness structure after repair (Kim et al., 2013). Due to its chemical and thermal stability, BC can be sterilized in easier and more effective ways than many other biological origin macromolecules; moreover, it resists in chemically aggressive environments without resorption (Mandour et al., 2019). Very important for TM surgery is BC transparency. In fact, not only tissue regeneration can be visually observed, but also recurrence of middle ear pathology can be better monitored. BC has excellent mechanical properties, is flexible and resistant to traction, which allows good surgical positioning that ultimately leads to optimal acoustic behavior. Finally, BC can secure the middle ear from infections by virtue of a dense nanofibrillar surface and a ∼10-μm thickness, which prevent the permeation of secretory liquids across ear compartments (de Oliveira Barud et al., 2016; Aleemardani et al., 2020). Kim et al. (2013) fabricated a transparent BC patch with nanofibrils structure from *Gluconacetobacter xylinus*. Their BC patch had a thickness of 10.33 ± 0.58 μm, with a tensile strength of 11.85 ± 2.43 MPa, and Young’s modulus of 11.90 ± 0.48 MPa. *In vitro* results showed that TM cell proliferation and migration were significantly stimulated with the presence of the BC patch. Interestingly, TM regeneration was successfully validated also in an animal model with traumatic TM perforations. The quality of healed TMs was also investigated and the three different layers of TM were successfully regenerate. Even though TMs with BC patches showed more irregular structures than controls, denser collagen fibers were obtained in the outer radial fibrous layers of TMs, which gave rise to a more effective sound conduction. Silveira et al. (2016) conducted a randomized controlled trial in which 40 patients with TM perforation, secondary to COM, were treated with temporal fascia or with BC. Different factors including surgical time, epithelialization time, perforation healing rate, hospital stay, and costs were evaluated. While the healing was similar in both groups, the authors highlighted the lowest average surgical time for the BC group (i.e., 14.06 min) *vs.* the fascia group (i.e., 76.50 min). This difference led to a remarkable cost reduction (i.e., −92%), as evaluated in the Brazilian public health system. In another study, Biskin et al. (2016) used BC membrane in myringoplasty for 12 patients (total 16 ears) and followed-up their postoperative condition in a 6–24-month range. At 6-month follow-up, TM perforation was completely closed in 13 ears, with neither granulation tissue formation nor infection, while it persisted in 3 ears. Thus, BC demonstrated to be safe and effective in small TM perforations. Differently, concerning large TM perforations, further investigations should be performed with more subjects and a long-term follow-up. Mandour et al. (2019) studied the efficiency of BC *vs.* fat and temporalis fascia grafts (the latter as a control) in 120 patients undergoing myringoplasty due to small or moderate size perforation. Their results demonstrated the efficiency of BC in repairing TM perforations, since the healing of small and moderate perforations occurred in shorter times with respect to the other grafts tested.

**FIGURE 2:**
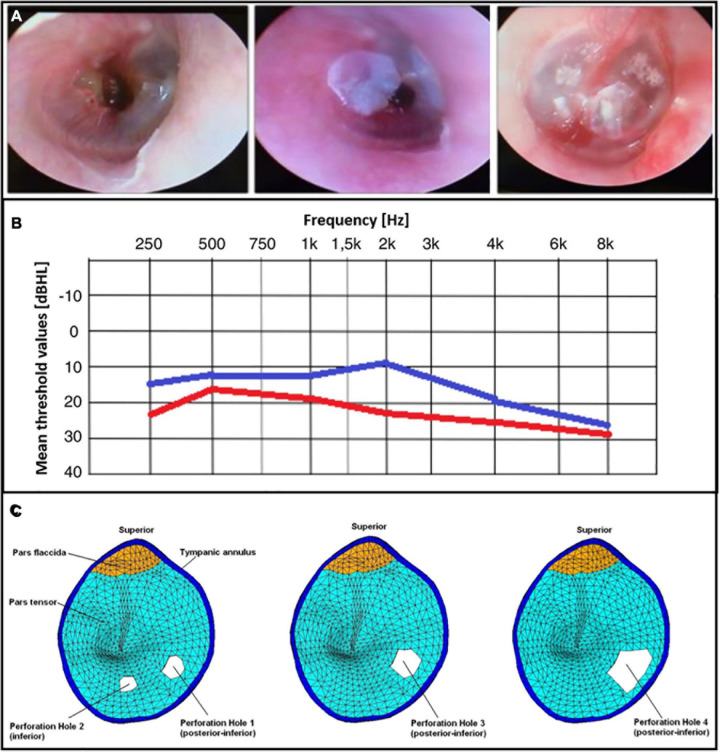
**(A)** Patients’ otoscopy procedures after the BC film placement. Reprinted with permission. **(B)** Tonal audiometry that compares the means values before (in red color) and after (in blue color) a BC placement across frequencies (de et al., 2020) © Creative Commons Attribution License, 1969, Elsevier. **(C)** Perforations of the TM modeled with a FEM. Reprinted with permission (Gan et al., 2009) © 2009 Acoustical Society of America.

Finally, de et al. (2020) used a BC film (Bionext^®^) in 24 TM traumatic perforations and carried out otoscopy and audiometric analyse. They observed an immediate symptomatic and functional recovery across the assessed patients (Figure 2A), as autophonia, tinnitus and ear fullness decreased. The application of a BC film also significantly improved the mean threshold values at all frequencies, with the exception of 8000 Hz (Figure 2B).

## Modeling Tools for BC and TM

*In silico* approaches can perfectly complement and ideally replace expensive experimental campaigns for optimizing material properties of interest for a specific application. In this section, we discuss the main state-of-the-art studies on modeling the BC and TM.

Molecular dynamics (MD) models have been a strategic tool to investigate the crystalline structure and properties of cellulose. A large number of force fields have been successfully employed for MD models including CHARMM, MARTINI, COMPASS (Zhou et al., 2020). MD models have addressed different levels of complexity of cellulose structure: (a) cell walls with little or no impurities with typical dimensions of micrometers (Zhou et al., 2017); (b) cellulose nanofibrils (CNFs) with diameters up to the nanometer scale; (c) cellulose nanocrystals (CNCs) that are fully at the nanoscale. Cellulose microfibrils, with diameters of about 35 A, are made of strands of pure cellulose chains with both crystalline and amorphous parts (Manley, 1964), with the latter that separates crystalline regions of different fibrils (Eichhorn, 2012). Cellulose has remarkable mechanical properties along the fibril direction that is due to its molecular structure made of rich hydroxyl groups and a diffused H-bond network that stabilize the crystalline assembly (Keten and Buehler, 2010). Covalent bonds connects the monomers along the fibril direction, giving a high directional stiffness (∼100 Gpa) and strength (Wohlert et al., 2012; Saito et al., 2013; Youssefian and Rahbar, 2015). Fracture of cellulose upon tensile loads has been studied with reactive models that unveiled the cause of rupture in the breaking of the C1-O4-C4 covalent bonds, identified as the strengthening agent of the fibrils. Laterally, because of the absence of such rigid connections, mechanical properties and probability of fracture are much lower (Wu et al., 2014). However, as it was described by Sinko et al. (2014) in their work, lateral dimensions play a significant role in fracture that is activated whenever the intermolecular interactions overcome a critical dimension (≈5–7 nm), which is close to the cellulose fibril dimension. Cellulose Iα is less stable upon temperature increase than cellulose Iβ because of the disposition of the glucose residues that, in the specific case of Iα are not parallel to the (200) plane (Hardy and Sarko, 1996). Concerning the interaction of cellulose with water, it has been seen that cellulose-based materials swell but not dissolve in water without any significant change in the structure of the crystals (Mazeau and Rivet, 2008).

Finite-Element Models (FEMs) have been a strategic tool to predict the macroscale behavior of cellulose. A first attempt to study fibrous cellulose networks was made by Rigdahl et al. (1984), who reported the stress distribution in a fibrous network highlighting the similarities with short-fiber reinforced composites. Moreover, the elongation capabilities of the constructs were found linearly dependent on the fiber density in contrast to the high stiffness of a single fiber. Moreover, they speculated that bond stiffness does not influence significantly the material properties of the network (Rigdahl et al., 1984). More recent studies, confirming these preliminary results were presented by Kulachenko et al. (2012). The most recent and interesting work was published by Mao et al. (2017). They developed a FEM of a cellulose fibrous nanopaper complementing the consolidated results at the state of the art. First, they predicted an elastic modulus of about 12 GPa, confirming also experimental data from earlier works (Henriksson et al., 2008; Retegi et al., 2010; Sehaqui et al., 2010; Chun et al., 2011; Mtibe et al., 2015). The elastic modulus is also dependent by the length of the fibers that, in turns, increases the inter-fiber connections promoting the stress distribution in the networks. In contrast, the diameter of the fibers plays the opposite effect because it affects the density of the assembly. As reported above, this latter parameter has a strong influence on the elasticity of the network that is reduced to zero with relative network densities below 0.39, equivalent to a 61% porosity of the assembly (Mao et al., 2017).

Tympanic membrane is undoubtedly the most interesting component of the middle ear to model. Compared to the other parts, the eardrum possesses a double peculiarity in terms of topology and material properties. A recent study by De Greef et al., proposed a further simplification in the definition of the mechanical parameters to describe the orthotropic model, limiting the input data to the three Young’s Moduli and a reference Poisson ratio usually set at 0.3. As for the damping parameter, it concluded that a constant isotropic loss factor (η) sufficed for predicting the TM viscoelastic behavior without further complicating the model (De Greef et al., 2017).

Perforations of the TM are an interesting subject of investigation from a modeling standpoint. Models can help to predict the behavioral effects of such alterations on the energy transfer efficiency and the following dysfunctions. FEMs have been used to overcome the limitations of circuit models (Voss et al., 2001, 2007) which do not include the structural features that have, instead, a significant effect on the outcomes (i.e., position and dimensions of the perforations). Gan et al. (2007) compared the results from temporal bones and their FEM, studying the effect of four different *pars tensa* perforations (Figure 2C). The first case concerned two small holes with areas in the order of 1 mm^2^ and diameter of about 1 mm. Secondly, they used a single larger perforation in the same site of the previous ones to understand the difference between the use of two holes vs. a larger hole even tough, the position may affect the results. Finally, a larger hole with a diameter of 2.35 mm and area of about 4 mm^2^ was modeled. Perforations affected the middle ear transfer function (i.e., velocity of the stapes over input pressure) mainly at low frequencies. Displacements of both the umbo and the stapes were reduced, being directly correlated with the hole dimensions on the TM. Moreover, the presence of multiple holes against perforations shifted the displacement curve toward the high frequencies (Gan et al., 2009).

## Conclusion

Biomaterials for repairing large TM perforations with optimal functional outcomes are still a challenge in otology. Revisiting biological biomaterials, like BC, for TM repair empowered by computational modeling offers novel strategies to accomplish this unmet clinical need. In particular, we believe that an integrated model that included the biological activity of BC would improve the understanding of the healing phenomena, being also a valuable tool for applications not limited to the TM, but also applicable to other scenarios in tissue engineering.

## Author Contributions

BA and MM collected the literature, designed the figures, and wrote the draft of the manuscript. BA, MM, and SD edited the manuscript format. SD revised the manuscript, edited the final version, and provided the funding support. All authors contributed to the article and approved the submitted version.

## Conflict of Interest

The authors declare that the research was conducted in the absence of any commercial or financial relationships that could be construed as a potential conflict of interest.
